# Supplementary material for: Impedance Iterative Learning Backstepping Control for Output-Constrained Multisection Continuum Arms Based on PMA
Source: Micromachines (Basel). 2022 Sep 16;13(9):1532. doi: 10.3390/mi13091532 (PMC9506357; doi:10.3390/mi13091532)

使用ANSYS经典版，导入一个管，进行划分网格，然后上下两端点分别用mass21进行刚性区域耦合好，如下：


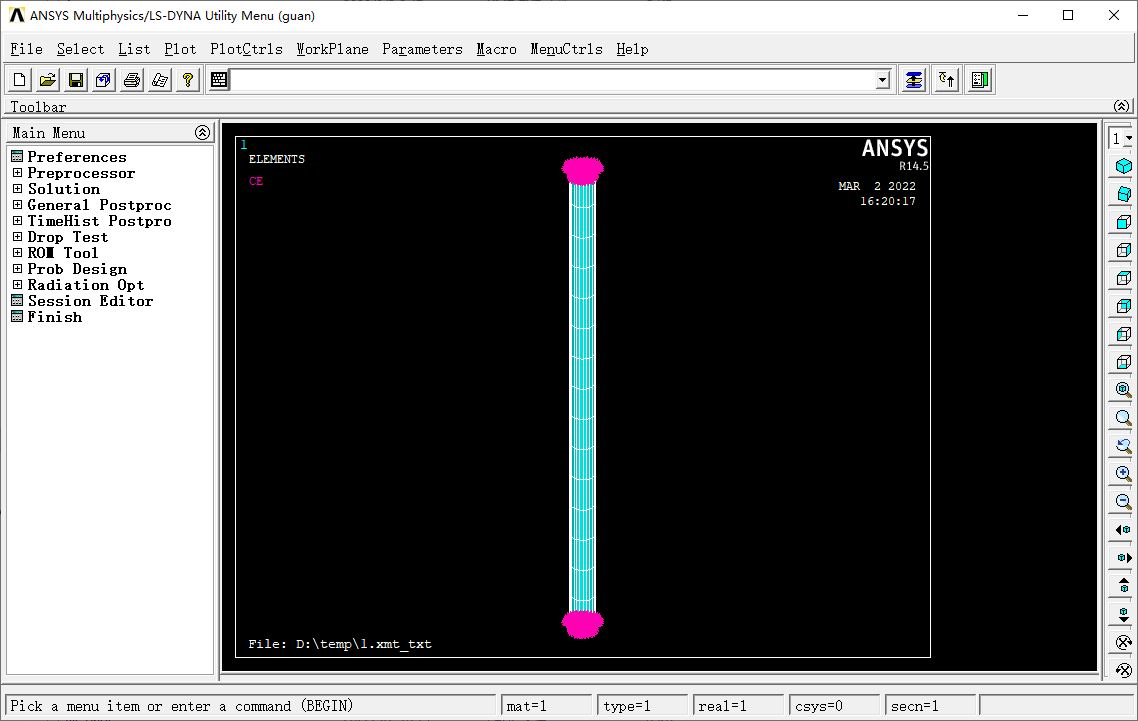


模型如上


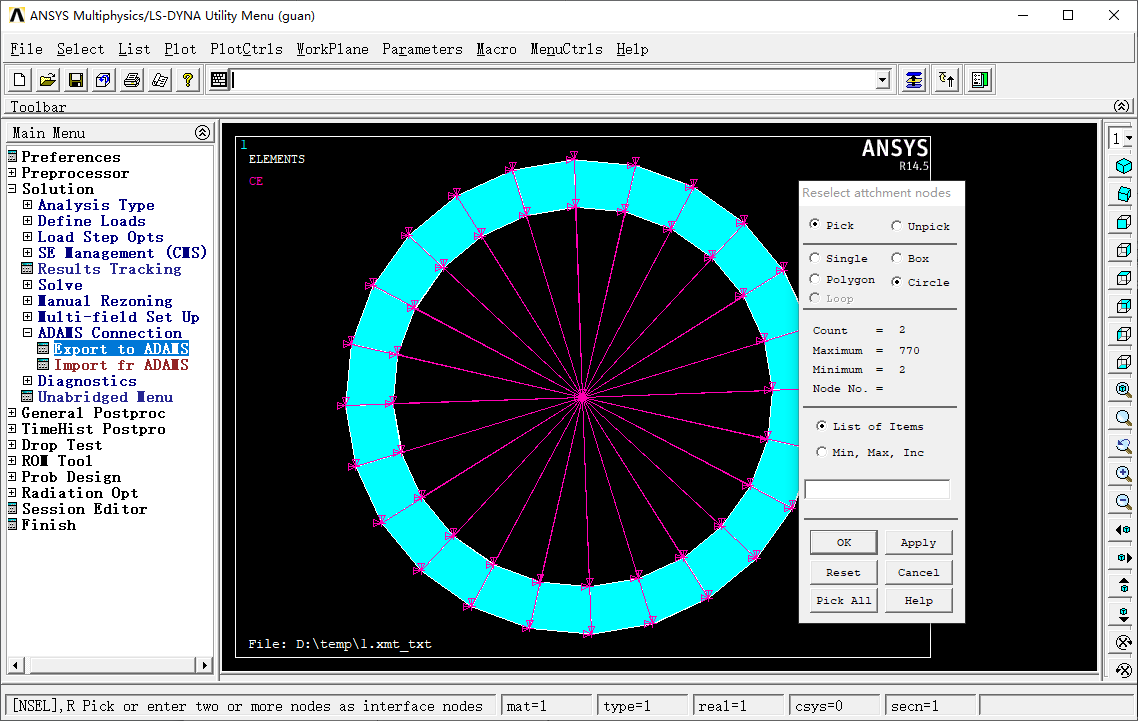


点击进行export to adams，然后选择中间的刚性节点。点击OK


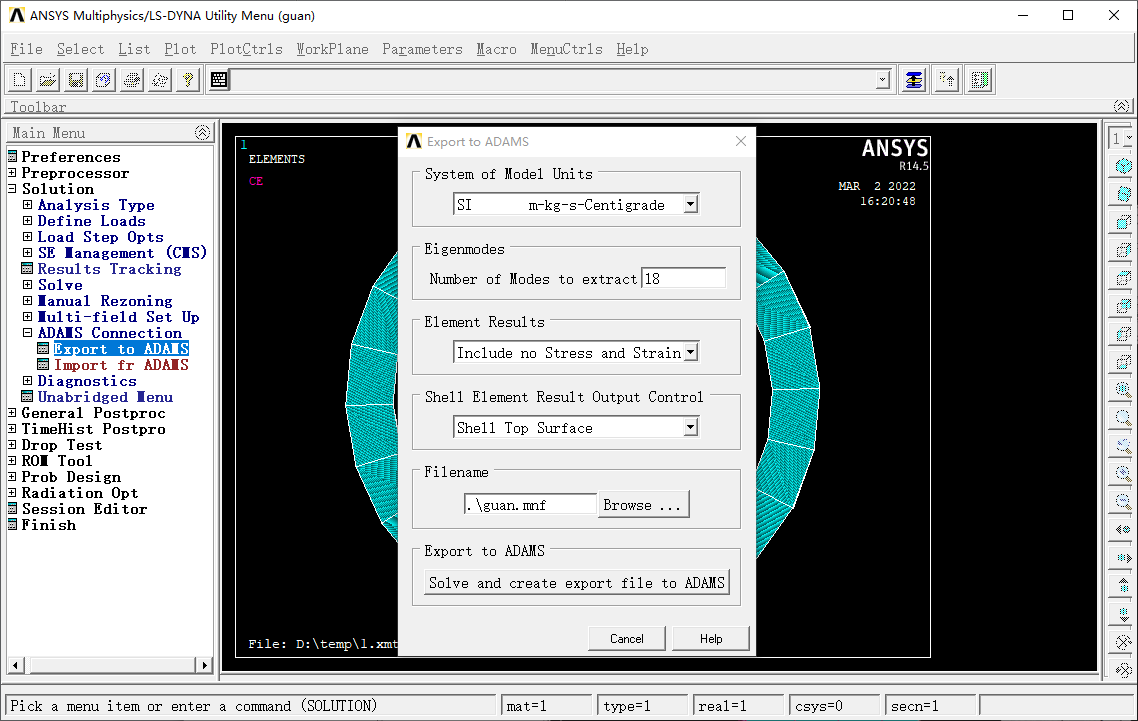


弹出如上，修改输出18阶，点击OK。就会生成柔性中性文件。

然后在adams中建立柔性体导入这个mnf，然后通过拷贝和移动生成其他软管。然后锁头连接软管和钢结构。建立连接软管两点的力。

建立各个软管两点的联合仿真变量。并修改各个力公式。这里略截图，与之前做的多刚体建立联合变量方法相同。然后导出Control_Plant_1.m（在ADAMS导出界面需要勾选静平衡）。这一步你也需要重新生成。


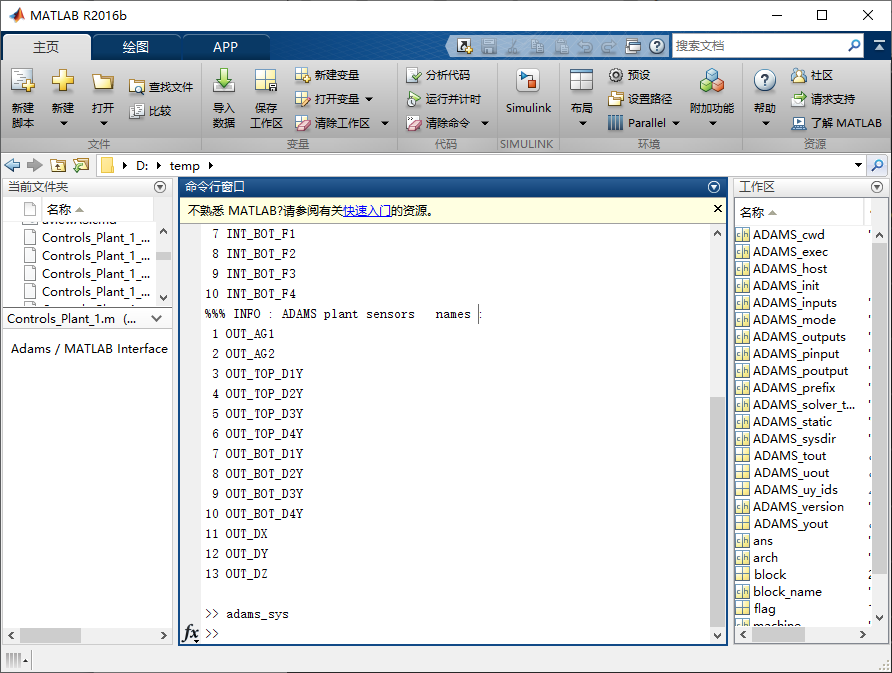


matlab进入到目录里，运行如上


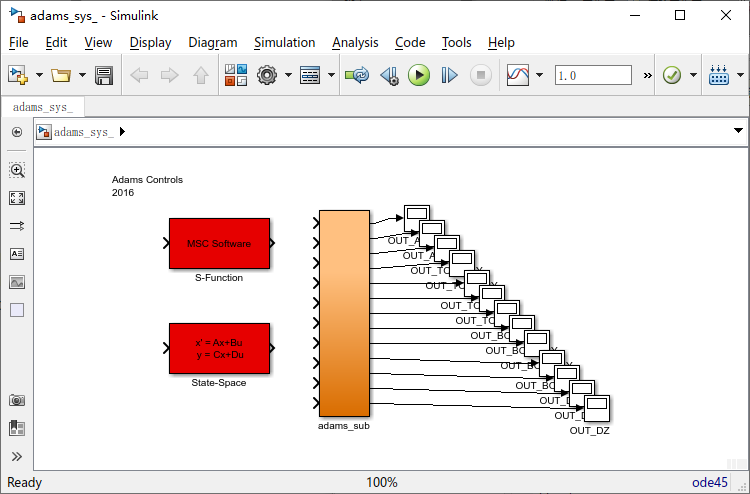


弹出如上，一共10个PID控制变量。生成这个替换做好的simulink模型，如下的橙色模块


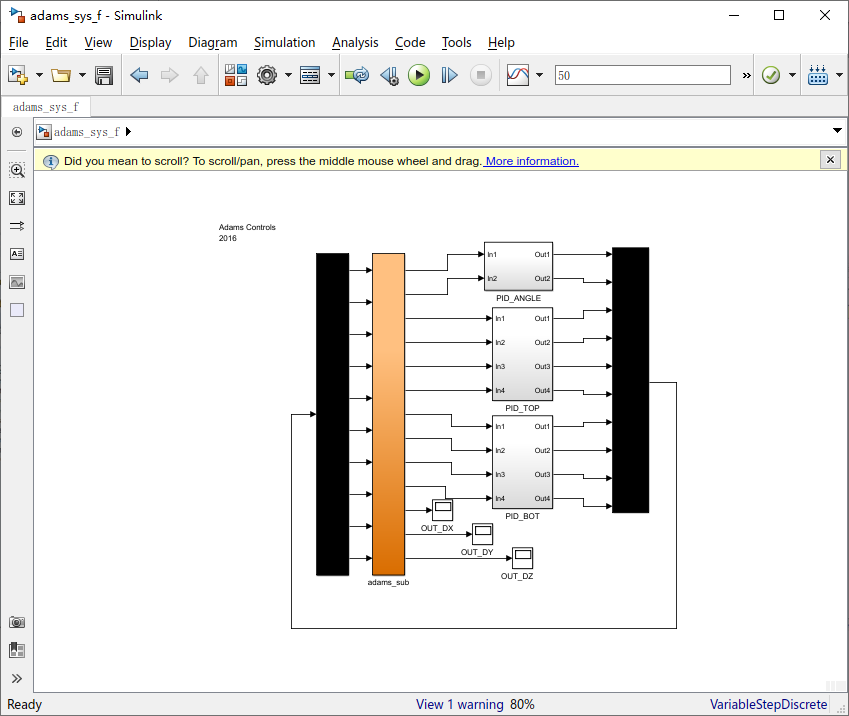


该模块与多刚体的类似。只不过把PID控制放在subsytem里面建立。如下：


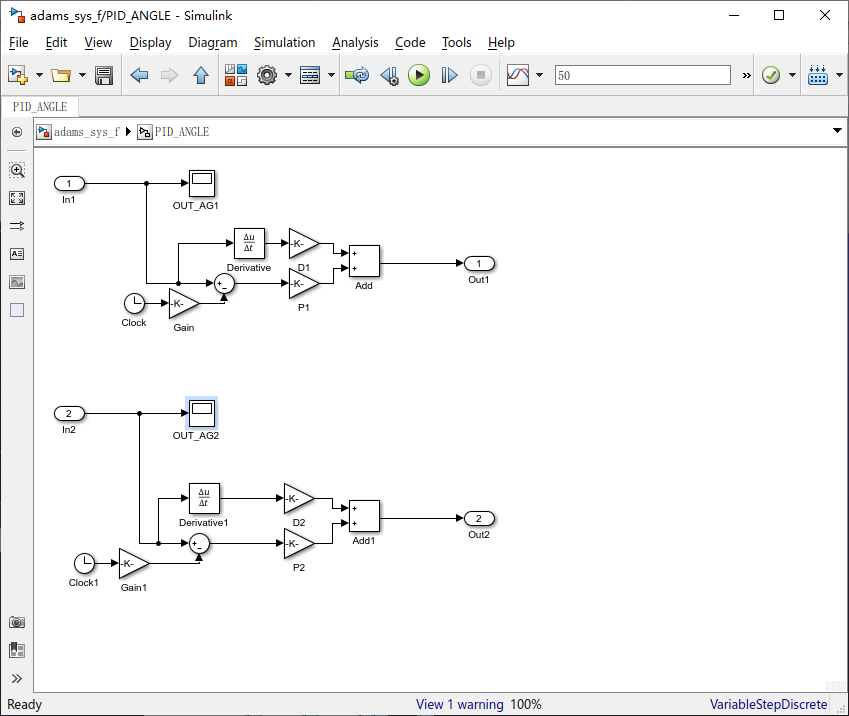


这个是PID控制两个转角。


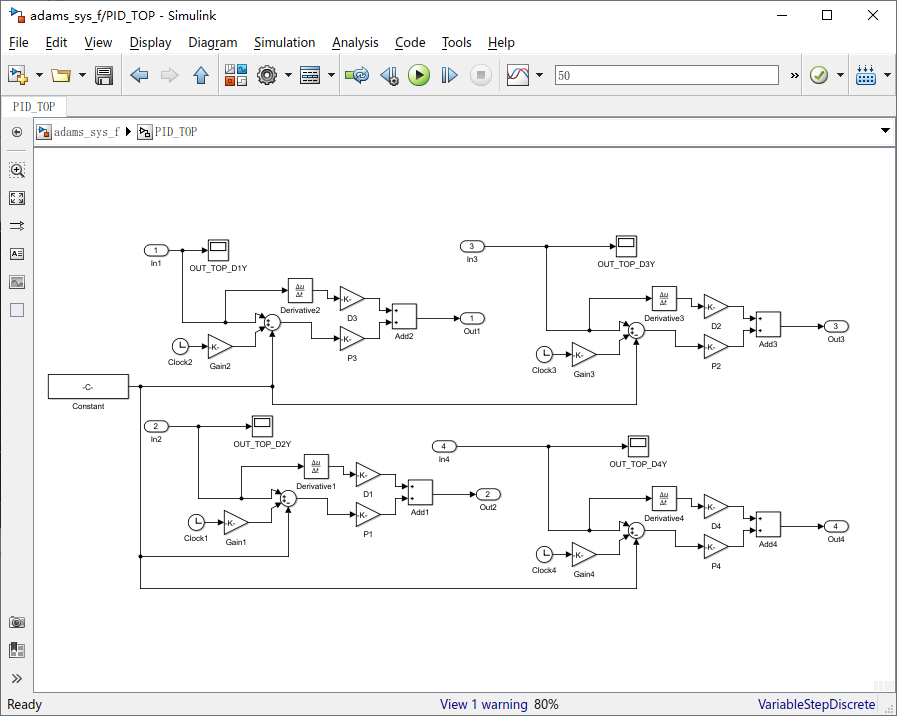


这个是控制上端的4个软管


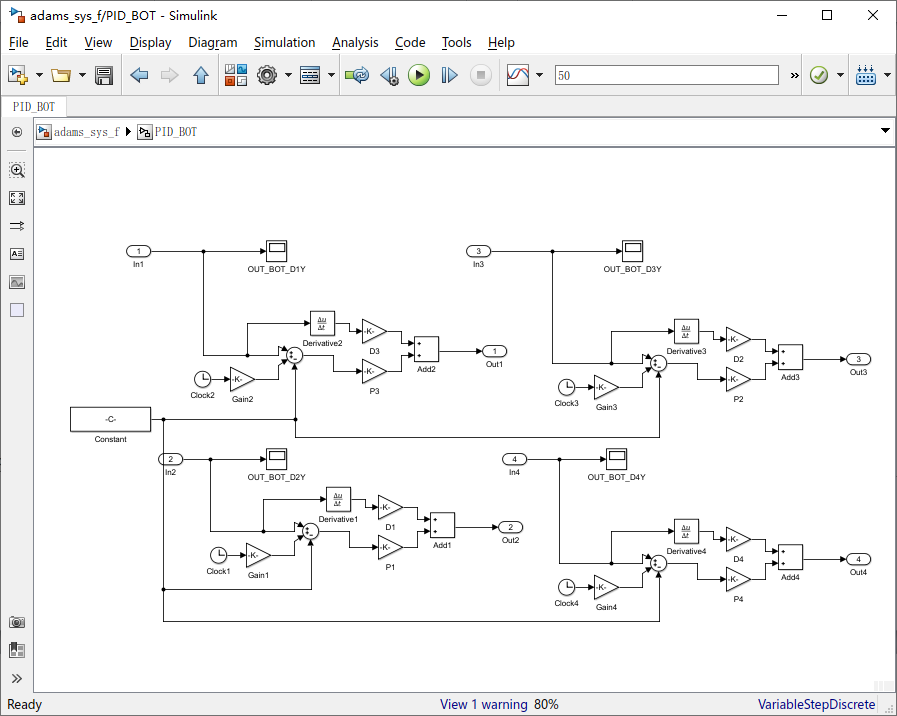


这个是控制下端的4个软管


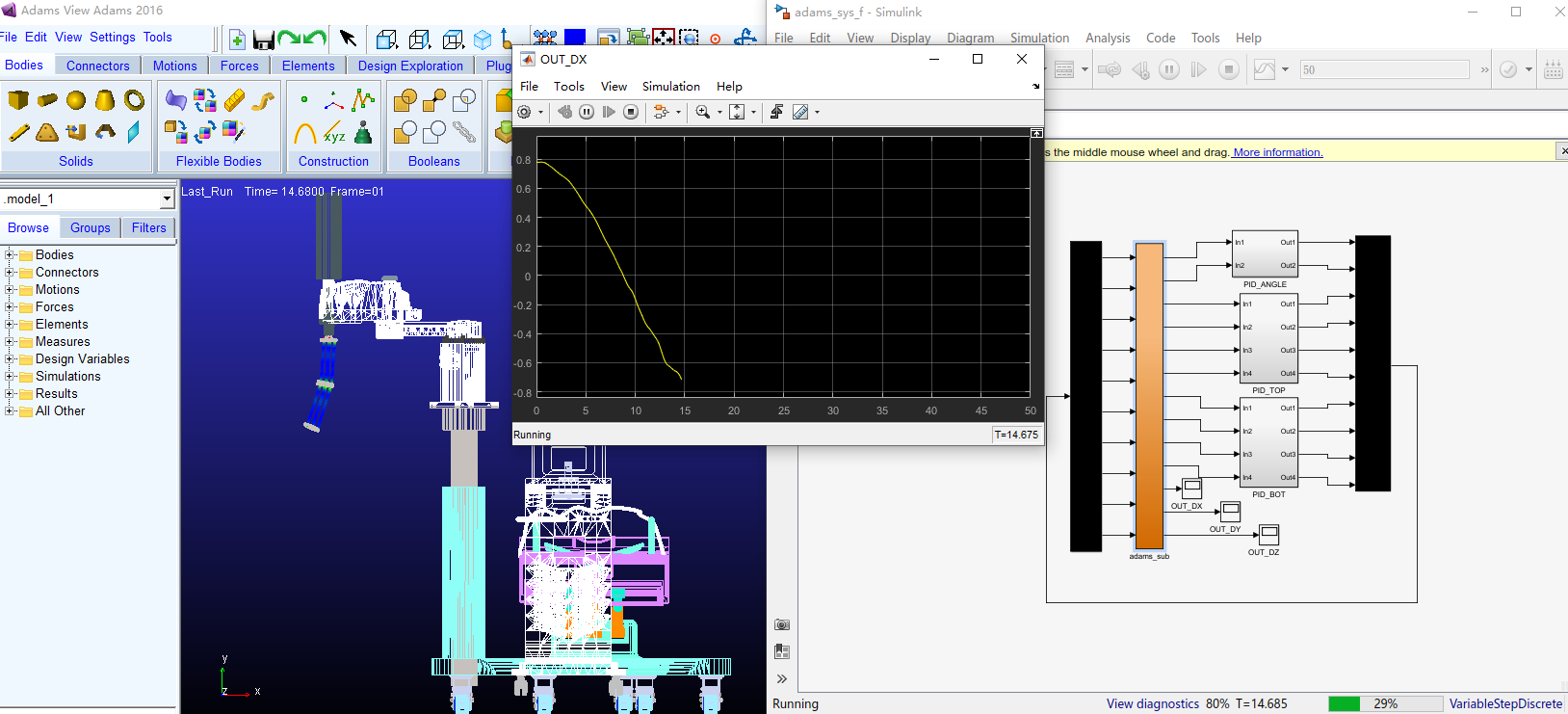


点击打开橙色、在点击红色模块，可以设置为交互模式，可以边联合边看动画过程。

注意由于是软管，这里simulink模块的仿真角度1和2转动速度太快（以前的5s转一圈）会导致软管飞起来，仿真失败。这里设置为50s转一圈。另外，软管的控制需要配合，PID管子的拉伸和压缩需要合理。

以下为仿真的部分结果：


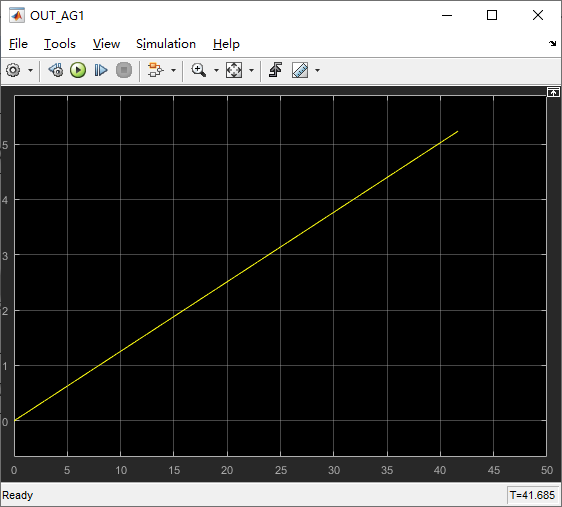


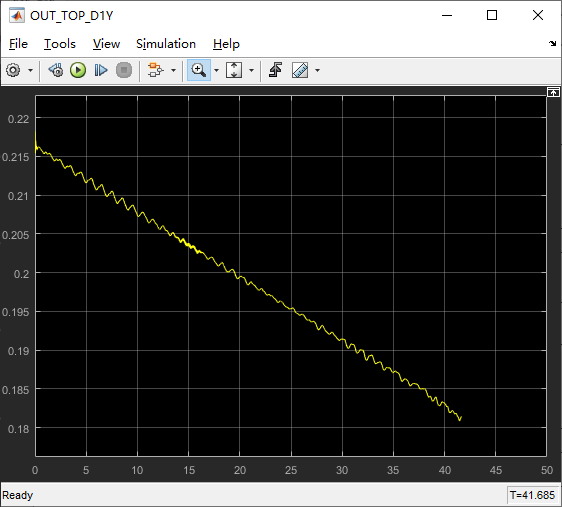


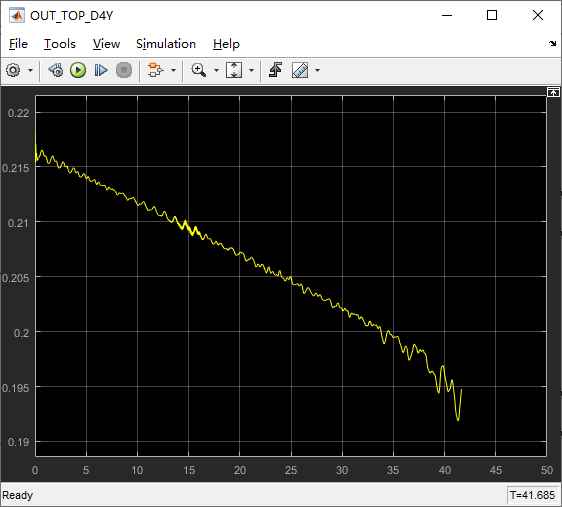


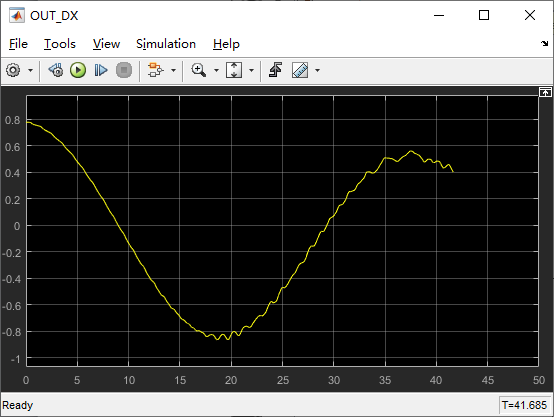


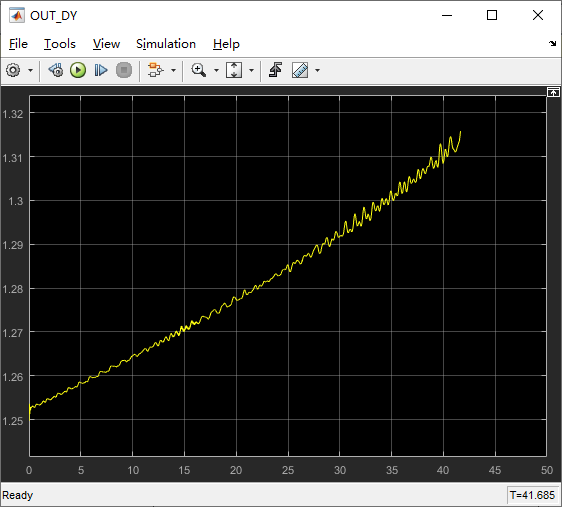


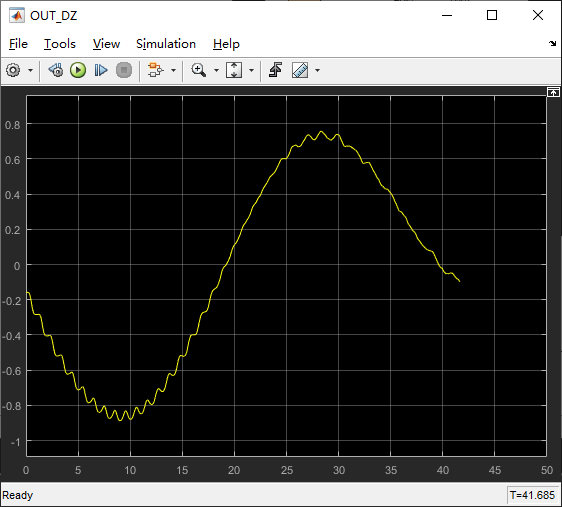

Supplement: Supplementary file 1 [file micromachines-13-01532-s001.zip › S2-Control Algorithm and Co-Simulation/S2-Control Algorithm and Co-Simulation/introduction.docx]
