# Supplementary material for: Impedance Iterative Learning Backstepping Control for Output-Constrained Multisection Continuum Arms Based on PMA
Source: Micromachines (Basel). 2022 Sep 16;13(9):1532. doi: 10.3390/mi13091532 (PMC9506357; doi:10.3390/mi13091532)
Supplement: Supplementary file 1 [file micromachines-13-01532-s001.zip › S2-Control Algorithm and Co-Simulation/S2-Control Algorithm and Co-Simulation/ANSYS and ADAMS operation details.doc]

Ansys和ADAMS柔性体转化问题的详细步骤

1. 进行单元类型定义，实体可选solid 45,质量单元选择mass21；
2. 编辑mass21质量单元preprocessor－&gt;real constant-&gt;add/edit/delete在对话框中填写属性，一般要很小的数值，如1e-5等；

3. 设置材料特性，要求有弹性模量（一般为2.06e11），泊松比（一般为0.3），密度（如钢为7800）这些参数；
4. 建立几何模型，使用solid 45进行划分网格，
5. 建立keypoints，此处注意，创建的keypoints的编号不能与模型单元的节点号重合，否则会引起原来的模型变形；

6. 选择mass21单元对5中建立的keypoints进行网格划分，建立起interface nodes，在导入adams后这些interface nodes会自动生成mark点，通过这些点和其他刚体或柔体建立连接；

7. 建立刚性区域（在ADAMS作为和外界连接的不变形区域，必不可少的），preprocessor－&gt;coupling/ceqn-&gt;rigid region,选择interface  nodes附近的区域的nodes与其相连，由于连接点的数目必须大于或等于2，所以刚性区域至少两个；先选择interface  node，单击Apply，再选周围的nodes。

8. 执行solution-&gt;ADAMS connection-&gt;Export to ADAMS命令，要选择的节点为7中建立刚性区域的节点（仅仅是interface  nodes），输出单位就选SI就行；即可生成*.mnf文件。

**不需要对任何节点作任何自由度的限制。**

**选择面时候用选择体里的面,然后选择连接接点,然后在连接接点内用BYLATION RESELET选择位置**

**注意警告数目一定要从新设置数目,不然自动退出.**

**附：catia导入ansys方法**

先将catia文件以model格式另存，打开ansys, file/import/catia…在打开的对话框中选择model格式的catia文件，就可以了。不要选file/import/catia v5。导入后只能见到线框，这个不影响以后的操作，不用管。单位也不需要做什么调整。

建立刚性区域的过程：

1.在有空洞的地方建立一个关键点，完成后，选择select/everything----entities后弹出

单击ok。

2.对实体进行网格划分，后对刚建的的关键点用mass21单元进行网格划分。通常list/node最后面得是该关键点划分后的node点，只有一个。

3完成后，选择select/everything----entities后弹出进行图示 单击ok。进行图示

，得到下图：

单击ok，后得到：

4 select/ entities后弹出，按照如图操作，点ok

后出现下图：

5.在选择建立的关键点网格划分后的此节点，如下图，

此处选择ALSO SELECT

Ok后

preprocessor－&gt;coupling/ceqn-&gt;rigid region,出现下图，此时先用single选择关键点网格划分后的节点，点apply，在用box选择周围面上的节点，点ok，

6，最后便形成刚性面。

**ansys和ADAMS柔性体转化问题的详细步骤**

多次看到有人问关于ansys和ADAMS的柔性体转化问题，经过本人的摸索实践，总结详细步骤如下：
从建立有限元模型后说起，进行了网格划分以后的步骤：
1.添加mass21质量单元preprocessor－>element type->add/edit/delete
选择add，添加mass21质量单元；
2.编辑mass21质量单元preprocessor－>real constant->add/edit/delete在对话框中填写属性，一般要很小的数值，如1e-5等
3.创建keypoints，preprocessor－>modeling->create->keypoints->in active Cs;此处注意，创建的keypoints的编号不能与模型单元的节点好重合，否则会引起原来的模型变形
4.选择mass21单元对3中建立的keypoints进行网格划分，建立起interface nodes；
5. 建立刚性区域（在ADAMS作为和外界连接的不变形区域，必不可少 的），preprocessor－>coupling/ceqn->rigid region,选择interface nodes附近的区域，由于连接点的数目必须大于或等于2，所以刚性区域至少两个
6.执行solution->ADAMS connection->Export to ADAMS命令，要选择的节点为5中建立刚性区域的节点

注意：1.材料属性是必不可少的
2.从ansys命令窗口输入/units,<name>
其中<name>-----SI.CGS.BFT和BIN四种单位中的一种，如果不是其中一种，则输入下面命令
/units，<L>,<M>,<T>,,,,<F>
L,M,T,F为用户单位和国际单位制（SI）之间的转换系数
如所用单位是mm，Mg（即吨），N，s，可输入
/units，user，1000，0.001，1，，，，1

# ANSYS导柔性体mnf到ADAMS心得

1.接口节点的选择有三种；

# a). 刚性区域法 在要建立接口节点的地方建立关键点（keypoint），并赋属性MASS21属性，mesh关键点，然后建立刚性区域； b). 蜘蛛网法        在要建立接口节点的地方建立节点（直接建立节点就行，没有必要用MASS21），用该节点与对应的节点创建梁单元（beam4），之所以称为蜘蛛网法，是因为一个主节点发散到周围的很多节点建立梁单元，看起来就像蜘蛛网一样; c). 梁单元法        在要建立接口节点的地方建立节点，然后用该节点与某一个节点创建一个梁单元。 三种方法中，第二种是是ANSYS优选的，第三种是不推荐的（我自己也没有试验过）。从图中看出第三种方法传递力的特性是比较差的，但是比第二种方法简单，不过要使用第二种方法的话，建议使用宏命令。第一种方法比较容易出错，刚性区域要反反复复地的建立删除，确实很烦人。 2. ansys报错： a). Unable to open file genCMS.mode.  Unit 46 is already in use.  Please    send your data to your ANSYS Technical Support Provider.   这个让ANSYS软件犯傻的问题，具体是怎么回事，怎样解决我也不知道。但是在我确信我的模型没有问题后，它还是这样报错，实在是没有办法了。有那找技术支持的时间，还不够重启软件一次ANSYS？重启之后果然没有这种错误了；不知这是解决方法，还是巧合。 b). Negative pivot encountered. This is  likely caused by insufficient displacement constraints or improper master dof selection to avoid rigid body motion of the superelement region currently selected     以及：superelement file subelem.sub is not available. 这种错误一般都是模型的连接有问题，特别是装配体，中间可能有自由的零部件，导致结构不能正常的传递力。另外就是模型中可能存在独立的节点（不在任何单元上的）； c). the number of modes expected (66)does not match the number found(61).Most likely, the model  has errors or there were more modes requested than exist. Please review the Prior modal  solution for warnings 这个问题应该先明白ANSYS中Number of Modes to exatract 中的值X 、选取的接口节点个数Y与ADAMS中看到的柔性性的模态数量Z的关系: Z=6×Y+X； 我的一个模型刚开始的时候我设置的X=6，选取了接口节点Y=8，然后就总是出现上面的报错，后面我减少了接口节点的个数，只选了三个，就没有报错了。这个方面我认为是能少选就少选，选得越多计算时间越长，越到高阶的模态对计算的结果影响也越少，到ADAMS中还得关闭一些高阶模态，所以能少则少！

三种接口节点的图片

[1.jpg](http://forum.simwe.com/attachment.php?aid=268468&k=657a0909eae4ed6d4fe19c09a67a3867&t=1292047106&fid=23&nothumb=yes&sid=93093iyXS9MPJYPWKUCBWUTOoRLOw6meyN6%2Fk1A0UeonUfc) *(14.78 KB)*

下载次数:1

2010-11-4 10:11

# 谢谢LZ,不过最后那个还是不怎么明白。。。出错的原因到底是因为模态数不够，还有那个转中性文件时，ANSYS是依据什么来提取模态呢？

# 理论上的错误应该不会有，我的理解是选择的节点太多，导致计算的矩阵过大或者容易产生奇异，在实际上没有计算完的时候就停止了，然后在下一步计算的时候有些模态没有在上一次的计算中提取出来，最后就报错了。 ANSY是怎样提取模态的理论依据我现在也没有弄明白。要是你知道了不妨上来交流交流，咱们讨论讨论。

# 还有就是模态正交化的时候可能不匹配
